# Inhibition of cyclin-dependent kinase 1 (CDK1) by indirubin derivatives in human tumour cells

**DOI:** 10.1054/bjoc.2000.1546

**Published:** 2001-01

**Authors:** D Marko, S Schätzle, A Friedel, A Genzlinger, H Zankl, L Meijer, G Eisenbrand

**Affiliations:** Department of Chemistry, Division of Food Chemistry and Environmental Toxicology, Department of Biology, Human Biology and Human Genetics, University of Kaiserslautern, Erwin-Schroedinger-Str. 52, Kaiserslautern, 67663, Germany; C.N.R.S., Cell Cycle Group, Station Biologique, B.P. 74, Roscoff cedex, Bretagne, 29682, France

**Keywords:** CDK1, cyclin B, indirubin, indirubin-3′-monoxime, cyclin-dependent kinase

## Abstract

The bisindole indirubin has been described, more than 30 years ago, as being clinically active in the treatment of human chronic myelocytic leukaemia. However, the underlying mechanism of action has remained unclear. We have reported previously that indirubin and its analogues are potent and selective inhibitors of cyclin-dependent kinases (CDK). In this study, we investigated the influence of indirubin and derivatives on CDK1/cyclin B kinase in human tumour cells at concentrations known to induce growth inhibition. Cells of the mammary carcinoma cell line MCF-7, synchronized by serum deprivation, after serum repletion stay arrested in the G_1_/G_0_ phase of the cell cycle in the presence of 2 μM indirubin-3′-monoxime. At higher drug concentrations (≥ 5 μM) an increase of the cell population in the G_2_/M phase is additionally observed. Cells synchronized in G_2_/M phase by nocodazole remain arrested in the G_2_/M phase after release, in the presence of indirubin-3′-monoxime (≥5 μM). After 24 h treatment with 10 μM indirubin-3′-monoxime a sub-G_2_ peak appears, indicative for the onset of apoptotic cell death. Treatment of MCF-7 cells with growth inhibitory concentrations of indirubin-3′-monoxime induces dose-dependent inhibition of the CDK1 activity in the cell. After 24 h treatment, a strong decrease of the CDK1 protein level along with a reduction of cyclin B in complex with CDK1 is observed. Taken together, the results of this study strongly suggest that inhibition of CDK activity in human tumour cells is a major mechanism by which indirubin derivatives exert their potent antitumour efficacy. © 2001 Cancer Research Campaign http://www.bjcancer.com


*British Journal of Cancer* (2001) **84**(2), 283–289
© 2001 Cancer Research Campaign
doi: 10.1054/ bjoc.2000.1546, available online at http://www.idealibrary.com on IDEAL®

### Inhibition of cyclin-dependent kinase 1 (CDK1) by indirubin derivatives in human tumour cells

**D Marko[1], S Schätzle[1], A Friedel[1], A Genzlinger[2], H Zankl[2], L Meijer[3] and G Eisenbrand[1]**

[1]Department of Chemistry, Division of Food Chemistry and Environmental Toxicology, [2]Department of Biology, Human Biology and Human Genetics, University of Kaiserslautern Erwin-Schroedinger-Str. 52, 67663 Kaiserslautern, Germany and [3]C.N.R.S., Cell Cycle Group, Station Biologique, B.P. 74, 29682 Roscoff cedex, Bretagne, France

**Summary** The bisindole indirubin has been described, more than 30 years ago, as being clinically active in the treatment of human chronic myelocytic leukaemia. However, the underlying mechanism of action has remained unclear. We have reported previously that indirubin and its analogues are potent and selective inhibitors of cyclin-dependent kinases (CDK). In this study, we investigated the influence of indirubin and derivatives on CDK1/cyclin B kinase in human tumour cells at concentrations known to induce growth inhibition. Cells of the mammary carcinoma cell line MCF-7, synchronized by serum deprivation, after serum repletion stay arrested in the $G_1/G_0$ phase of the cell cycle in the presence of 2 µM indirubin-3′-monoxime. At higher drug concentrations (≥ 5 µM) an increase of the cell population in the $G_2$/M phase is additionally observed. Cells synchronized in $G_2$/M phase by nocodazole remain arrested in the $G_2$/M phase after release, in the presence of indirubin-3′-monoxime (≥5 µM). After 24 h treatment with 10 µM indirubin-3′-monoxime a sub-$G_2$ peak appears, indicative for the onset of apoptotic cell death. Treatment of MCF-7 cells with growth inhibitory concentrations of indirubin-3′-monoxime induces dose-dependent inhibition of the CDK1 activity in the cell. After 24 h treatment, a strong decrease of the CDK1 protein level along with a reduction of cyclin B in complex with CDK1 is observed. Taken together, the results of this study strongly suggest that inhibition of CDK activity in human tumour cells is a major mechanism by which indirubin derivatives exert their potent antitumour efficacy. © 2001 Cancer Research Campaign http://www.bjcancer.com

**Keywords**: CDK1; cyclin B; indirubin; indirubin-3′-monoxime; cyclin-dependent kinase

In the traditional Chinese medicine, the preparation Danggui Longhui Wan, consisting of 11 herbal medicines, has been used for the treatment of various chronic diseases (Chinese Pharmacopoeia, edition 1995, Vol. 1). Danggui Longhui Wan was shown to be clinically active in chronic myelocytic leukaemia (CML) (Institute of Haematology, Chinese Academy of Medicinal Sciences, 1979). The antileukaemic activity of the complex mixture was found to be associated with one ingredient, Qing Dai (*Indigo naturalis*), containing the well known blue dye indigo as the main constituent (Chen and Xie, 1984). Further investigations revealed that the antileukaemic principle was not the 2,2′-bisindole indigo, but could be traced to the minor constituent indirubin, a red colored 3,2′-bisindole isomer (Wu et al, 1979; Zheng et al, 1979). In clinical trials with patients suffering from chronic myelocytic and chronic granulocytic leukaemia, indirubin was reported to induce complete remission in 26% and partial remission in 33% of the 314 cases, showing only low toxicity and limited side effects (Cooperative Group of Clinical Therapy of Indirubin, 1980). Indirubin has been reported to inhibit DNA and protein synthesis in various cell lines (Wu et al, 1980). However, the underlying mechanism of action of indirubin has remained largely unknown.

We have recently discovered that indirubin and its derivatives are selective and potent inhibitors of cyclin-dependent kinases (Hoessel et al, 1999). CDK1/cyclin B kinase isolated from starfish oocytes is inhibited by indirubin with an $IC_{50}$ value of 10 µM. Indirubin-3′-monoxime exhibited a higher inhibitory potency with an $IC_{50}$ value of 0.18 µM. The water-soluble derivative indirubin-5-sulfonate was much more potent, exhibiting an $IC_{50}$ value as low as 0.055 µM (Hoessel et al, 1999). In the present study we investigated whether in human tumour cells cyclin-dependent kinases are affected by indirubin derivatives, to address the question whether this inhibition is relevant for the growth inhibitory properties of these compounds.

*Received 3 April 2000*
*Revised 8 September 2000*
*Accepted 20 September 2000*

*Correspondence to:* D Marko

### MATERIALS AND METHODS

### Chemicals

Indirubin, indirubin-5-sulfonate and indirubin-3′-monoxime were synthesized as described previously (Hoessel et al, 1999). Olomoucine (2-(2-hydroxyethylamino)-6-benzylamino-9-methylpurine) and nocodazole (methyl-[5-(2-thienylcarbonyl)-1H-benzimidazol-2-yl]-carbamate) were purchased from Sigma (Deisenhofen, Germany). All compounds were dissolved in dimethyl sulfoxide (DMSO)

### Cell culture

The human mammary carcinoma cell line MCF-7 and the large cell lung tumour xenograft cell line LXFL529L (Fiebig et al, 1992) were cultured in RPMI 1640 medium with 10% fetal calf serum (FCS) and 1% penicillin/streptomycin in humidified incubators (37˚C, 5% $CO_2$). Cell culture medium and supplements were obtained from Gibco Life Technologies (Karlsruhe, Germany). Cells were routinely tested for the absence of mycoplasm contamination. For synchronization, cells at a density of 60–70% were held for 48 h in RPMI 1640 medium without FCS. As an alternative synchronization method, cells were treated with 0.5 µg ml$^{-1}$ nocodazole for 16 h. Cells were released from the block by removal of the respective medium, careful washing with phosphate buffered saline (PBS), and by adding serum repleted medium containing drug or vehicle.

### Sulforhodamine B assay

Effects on cell growth were determined as described previously (Skehan et al, 1990). Briefly, cells were seeded into 24-well plates and allowed to grow for 24 h before treatment. Thereafter, cells were incubated with the respective drug for 3 days in serum-containing medium. Incubation was stopped by addition of trichloro acetic acid. After 1 h at 4˚C, plates were washed 4 times with water. The dried plates were stained with sulforhodamine B. The dye was eluted and quantified photometrically at 570 nm. Cytotoxicity was determined as percent survival, determined by the number of treated over control cells × 100 (% T/C).

### Immunoprecipitation

For immunoprecipitation, synchronized cells (1 × 10$^{7}$) were washed with PBS and lysed at 4˚C in 0.5 ml lysis buffer (10 mM Tris, pH 7.4, 1 mM EDTA, 1 mM EGTA, 150 mM NaCl, 0.2 mM PMSF, 0.2 mM sodium vanadate, 1% Triton ®X-100, 0.5% Nonidet P-40). After centrifugation (10 min, 4˚C, 10 000 ***g***), the lysate, adjusted to 400 µg protein, was incubated for 1 h at 4˚C with a mouse monoclonal antibody raised against human CDK1 (anti-CDK1; Dianova, Hamburg, Germany). The immune complex was absorbed to protein G-coupled sepharose beads (Sigma, Deisenhofen, Germany) for 1 h at 4˚C. Thereafter, the beads were washed 3 times with PBS.

### Western blotting

Immunoprecipitated proteins were eluted from the sepharose beads by addition of 1% SDS and loaded onto a 12% SDS-polyacrylamide gel.

For determination of the total amount of CDK1 or cyclin B, MCF-7 cells were grown onto 10 cm Petri dishes for 24 h and subsequently synchronized by treatment with nocodazole for 16 h (0.5 µg ml$^{-1}$). Thereafter, the plates were washed with PBS and incubated with indirubin-3′-monoxime (5 µM) or the solvent DMSO (final concentration 1%) for control. For every time point (1 h, 3 h, 6 h and 24 h) one plate treated with indirubin-3′-monoxime and one of the DMSO treated plates were harvested and processed in parallel. The plates were washed with PBS and than harvested in 500 µl RIPA buffer (consisting of 150 mM NaCl, 1% Nonidet P-40, 0.1% SDS, 0.5% sodium deoxycholate, 50 mM Tris-HCl, pH 7.5, 25 mM benzamidine, 0.5 µg ml$^{-1}$ leupeptin, 0.7 µg ml$^{-1}$ pepstatin, 2 µg ml$^{-1}$ aprotinin, 5 mg ml$^{-1}$ trypsin soybean inhibitor, 2 mM phenylmethylsulfonyl fluoride, 50 mM NaF), homogenized and centrifuged for 10 min (4˚C, 10 000 ***g***). The supernatants (cell lysate) were adjusted with respect to protein content, mixed with the respective amount of 6-fold loading buffer and directly subjected to electrophoresis on a 12% SDS-polyacrylamide gel.

After electrophoresis the proteins were transferred onto a nitrocellulose membrane. After blocking with 4% bovine serum albumin, membranes were incubated with the respective monoclonal antibody. For determination of the total cellular content of CDK1, a mouse monoclonal antibody against human CDK1 (anti-CDK1; Dianova, Hamburg, Germany) was used. The total cellular level of cyclin B and the amount of cyclin B in complex with CDK1 were determined using a mouse monoclonal antibody against human cyclin B (anti-cyclin B; Dianova, Hamburg, Germany). The membranes were treated with the antibody solution for 1.5 h at room temperature. The membranes were washed 3 times in TBST buffer and incubated with anti-mouse IgG (H+L) peroxidase conjugate (Roche Diagnostics, Mannheim, Germany) for 1 h at room temperature. The protein bands were detected by enhanced chemoluminescence (ECL, Amersham Buchler GmbH, Braunschweig, Germany), using a Lumi-Imager™ with the LumiAnalyst 3.0 software for quantification (Roche Diagnostics, Mannheim, Germany). Each experiment was performed three times (different cell passages) and the values are given as mean ± SE.

### Histone H1 kinase assay

Histone H1 phosphorylation, as a measure for CDK1 activity, was assayed using a kit from Biozol (Eching, Germany). Immunoprecipitated CDK1 was resuspended in 10 µl assay dilution buffer (20 mM MOPS, pH 7.2, 25 mM β-glycerol phosphate, 5 mM EGTA, 1 mM sodium orthovanadate and 1 mM DTT), 20 µg of histone H1 (Biozol), 10 µl inhibitor cocktail (Biozol), 10 µl of [$^{32}$P]-ATP-Mixture (75 mM magnesium chloride, 500 µM ATP in assay dilution buffer) and 100 µCi [$^{32}$P]-ATP (3000 Ci/mmol, Hartmann Analytics, Braunschweig, Germany). The kinase reaction was performed for 15 min under shaking at 30˚C. Following a brief centrifugation, the supernatant (25 µl) was applied to phosphocellulose sheets, which have been rinsed 5 times with 1% phosphoric acid. Radioactivity was determined using a liquid scintillation counter.

### Cellular uptake

LXFL529L cells seeded into 10 cm Petri dishes were cultivated for 24 h. Thereafter, they were incubated for 2 h with the respective compound in serum-containing medium. For each concentration two Petri dishes were treated in parallel. At the end of the allotted time, substance-containing medium was removed and the Petri dishes were washed twice with PBS. Cells were harvested by scraping in Tris-buffer (50 mM Tris-HCl, pH 7.4, 10 mM $MgCl_2$, 0.1 mM EDTA) and cracked by 3 cycles of freezing and thawing in liquid nitrogen. Centrifugation of the homogenate for 15 min at 10 000 ***g*** yielded the cytosolic fraction in the supernatant to be analysed separately. The remaining pellet was treated with Tris-buffer containing 0.5% Triton® X-100 for solubilization of the membrane proteins. The protein content of the samples was determined according to the method of Bradford. Subsequently, precipitation of the proteins and elution of the test compounds was performed by addition of methanol (–20˚C). After centrifugation for 15 min at 10 000 ***g*** the supernatant was applied to HPLC.

HPLC determination of indirubin and indirubin-3′-monoxime was performed on a reversed phase column (RP 18 endcapped, LiChroCART 125–4, Merck, Darmstadt, Germany) with UV detection at 292 nm, using methanol/$H_2O$ (70/30) at 1 ml min$^{-1}$. Indirubin-5-sulfonate was determined using an aqueous solution of tetrabutylammoniumbromide (5 mM) and methanol (40/60) as mobile phase. Quantification was done by comparison with external standards. Each sample was analysed at least twice. For each concentration two Petri dishes were treated and analysed in

 © 2001 Cancer Research Campaign parallel. The whole experiment was performed 3 times. The values are given as mean ± SD.

### Flow cytometry

Cells were seeded into 10 cm Petri dishes and treatment started at a cell density of 60–70% (logarithmical growth). After synchronization and drug treatment, cells were trypsinized and centrifuged at 200 ***g***. The cell pellet was washed with PBS and fixed in 70% ethanol (4˚C). Flow cytometry was performed as described previously (Marko et al, 1998).

### RESULTS

### Growth inhibition

Indirubin, indirubin-3′-monoxime and indirubin-5-sulfonate (Figure 1) were tested for growth inhibitory activity using the human large cell lung carcinoma line LXFL529L and the human mammary carcinoma cell line MCF-7 (Table 1). In LXFL529L cells indirubin-3′-monoxime was clearly more active than indirubin, however, no significant difference in growth inhibition was detected between the two compounds on MCF-7 cells. Indirubin-5-sulfonate, exhibiting the highest potency for in-vitro inhibition of CDK1/cyclin B kinase from starfish oocytes (Hoessel et al, 1999), was inactive up to 100 µM in both cell lines.

### Cellular uptake and subcellular localization of indirubins

To investigate potential causes for the discrepancy between the inhibition of isolated CDKs and growth inhibitory properties in cell culture, cellular uptake and subcellular distribution of the compounds was determined. LXFL529L cells were incubated with the respective compounds for 2 h and the cellular content was determined by HPLC using UV detection. Incubation with 10 µM indirubin resulted in a cellular content of 0.15 ± 0.08 µg $mg^{-1}$ protein, 93 ± 6% being localized in the particulate fraction. Incubation with 20 µM only marginally enhanced the cellular content to 0.20 ± 0.08 µg $mg^{-1}$ protein, with 93 ± 6% in the particulate. Indirubin and indirubin-3′-monoxime were taken up to a comparable extent. Incubation with 10 µM indirubin-3′-monoxime resulted in a cellular content of 0.16 ± 0.01 µg $mg^{-1}$ protein, but in contrast to indirubin a substantial proportion of indirubin-3′-monoxime was detected in the cytosol (43 ± 14%). Incubation with 20 µM indirubin-3′-monoxime only slightly enhanced the cellular content (0.23 ± 0.03 µg $mg^{-1}$ protein) with 44 ± 16% of the compound localized in the cytosol. Indirubin-5-sulfonate was not detectable at all in the cells even after incubation with concentrations as high as 50 µM.

Based on these results, indirubin-3′-monoxime, representing a potent inhibitor of isolated CDK1/cyclin B with an $IC_{50}$ of 0.18 µM (Hoessel et al, 1999) that showed the highest growth inhibitory efficacy was selected for the following mechanistic studies.

### Effects on cell cycle distribution and induction of apoptosis

Inhibition of cyclin-dependent kinases is expected to result in changes of the cell cycle distribution. Therefore, the effect of indirubin-3′-monoxime on the cell cycle distribution of MCF-7 cells was determined by flow cytometry. MCF-7 cells were synchronized by two different methods, to compare the effect of indirubin-3′-monoxime on cell populations with differential cell cycle distribution after release from synchronization (Figure 2, Table 2). Table 2 shows the differential effects of the two synchronization protocols used in this study and shows the different velocity by which the cells return to the unsynchronized cell cycle distribution. Serum deprivation of MCF-7 cells for 48 h induced a significant increase of cells in the $G_1/G_0$ phase of the cell cycle, as well as a slight increase of cells in $G_2/M$, concomitantly with a loss of cells in S-phase (Table 2). 6 h after release by serum repletion, control cells remained arrested in $G_1/G_0$, whereas already a reduction of cells in $G_2/M$ became apparent (Table 2). Cells treated with indirubin-3′-monoxime (20 µM) showed 6 h after release from

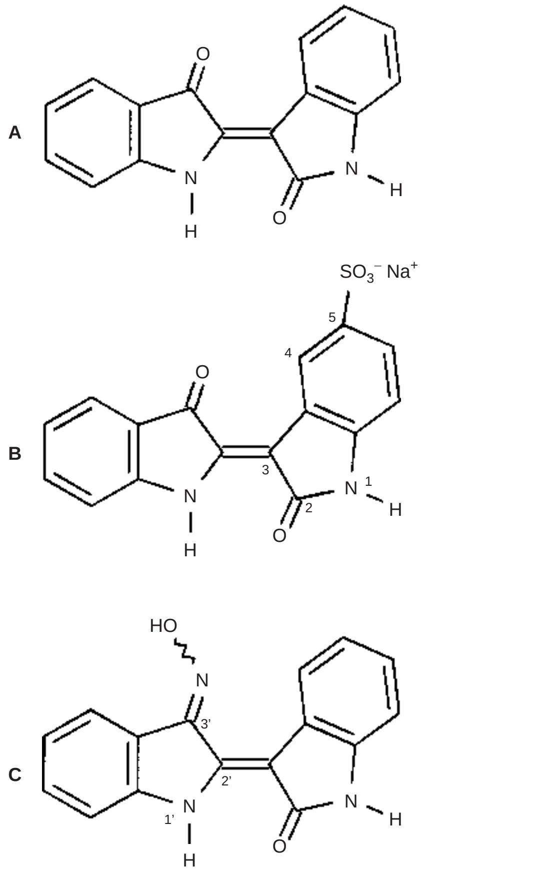

**Figure 1** (**A**) Indirubin, (**B**) indirubin-5-sulfonate (sodium salt), (**C**) indirubin-3′-monoxime

**Table 1** Growth inhibition of indirubin and derivatives in the sulforhodamine B assay

| Compound | Growth inhibition, $IC_{50}$ (µM)[a] | |
|---|---|---|
| | LXFL529L | MCF-7 |
| Indirubin | 9.9 ± 0.1 | 4.0 ± 2.0 |
| Indirubin-3′-monoxime | 3.0 ± 0.5 | 3.3 ± 0.4 |
| Indirubin-5-sulfonate | ≥ 100 | ≥ 100 |

[a]Growth inhibition was determined using the sulforhodamine B assay (Skehan et al, 1990), incubation time 3 d. $IC_{50}$ values were calculated as survival of treated cells over control cells × 100 (T/C%); values are given as mean ± SD of three separate assays, each done in quadruplicate.

© 2001 Cancer Research Campaign

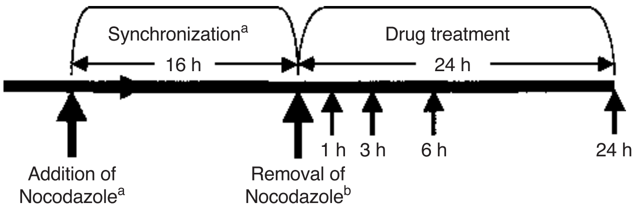

**Figure 2** Experimental design. [a]For the cell cycle experiments depicted in Figures 3A, B and 4 A–C serum depletion for 48 h was used as an alternative synchronization method. Cells were kept for 48 h in serum-free medium. [b]Thereafter, to release the cells from the block serum was repleted. At this time point, incubation with indirubin-3'-monoxime was started

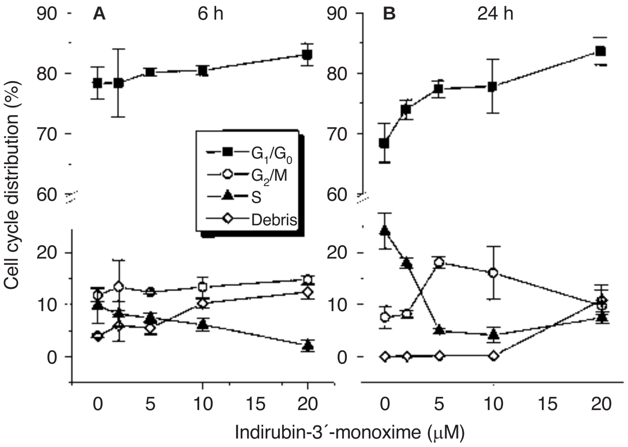

**Figure 3** Time- and concentration-dependent effect of indirubin-3′-monoxime on the cell cycle distribution of MCF-7 cells, synchronized by serum deprivation. Cells were treated for 6 h (**A**) and 24 h (**B**) with indirubin-3′-monoxime. After DAPI staining, cell cycle distribution was determined by flow cytometry. (■) $G_1/G_0$, (O) $G_2/M$, (▲) S, (◊) debris. For each concentration 2 Petri dishes were treated in parallel, the whole experiment was repeated twice. The values are given as mean ± SD

serum-deprivation a slight increase in $G_1/G_0$ (Figure 3A). 24 h after release control cells exhibited a 10% reduction in $G_1/G_0$ and an already normalized $G_2/M$ population (Table 2). In contrast, cells treated with 2 µM indirubin-3′-monoxime at the same time point after serum replenishment showed a significant increase in $G_1/G_0$. At concentrations ≥5 µM this effect was enhanced and additionally an increase of cells in $G_2/M$ was observed (Figure 3B; Figure 4A–C). Furthermore, at 10 µM indirubin-3′-monoxime a small sub-$G_1$-peak became apparent, indicating the onset of apoptotic cell death (Figure 4C). ELISA-based analysis of fragmented DNA supported these results, revealing an initial increase even at 5 µM indirubin-3′-monoxime, which was strongly enhanced at 15 µM (data not shown).

Treatment of MCF-7 cells with nocodazole induced a strong transient arrest in the $G_2/M$ phase of the cell cycle, concomitant with a marked depletion of the $G_1/G_0$ population (Table 2). In the untreated control, 6 h after withdrawal of nocodazole, the number of cells in $G_2/M$ had already dropped by approximately 50% and the cell number in $G_1/G_0$ had normalized (Table 2). In contrast, treatment with indirubin-3′-monoxime at concentrations ≥5 µM kept the cells in the $G_2/M$ phase and prevented an increase of the $G_1/G_0$ cells (Figure 4D–F, Figure 5A). In the untreated control, 24 h after withdrawal of nocodazole the number of cells in $G_2/M$ had dropped further, heading to normal cell cycle distribution (Table 2). In contrast, the presence of indirubin-3′-monoxime (≥5 µM) induced a strong arrest in the $G_2/M$ phase of the cell cycle at the same time point (Figure 4G–I, Figure 5B). In addition, at 10 µM indirubin-3′-monoxime a sub-$G_2$-peak became apparent (Figure 4I), indicating the onset of apoptotic cell death.

### Inhibition of CDK activity in MCF-7 cells

To investigate whether indirubin-3′-monoxime affects CDK activity in human tumour cells, we selected CDK1/cyclin B kinase as a representative target. MCF-7 cells were synchronized by nocodazole treatment and subsequently incubated with indirubin-3′-monoxime or the solvent DMSO (1%) for 24 h (Figure 2) in serum-containing medium comparable to the cell cycle experiments (Figures 4G–I and 5B). After immunoprecipitation with a monoclonal antibody raised against human CDK1, the phosphorylation of histone H1 was determined as a measure for CDK1 activity. Incubation with 5 µM indirubin-3′-monoxime induced a highly significant reduction of CDK1 activity ($P < 0.005$ compared to the control), an effect which was enhanced by incubation with 10 µM (Figure 6). In comparison, incubation with olomoucine was found to be less effective, inhibiting 50% of the kinase activity at 100 µM. In cells arrested by serum-deprivation, dose-dependent inhibition of the intracellular CDK1 activity by indirubin-3′-monoxime was observed to a similar extent (data not shown).

**Table 2** Effects of synchronization and release on the cell cycle distribution of otherwise untreated MCF-7 cells[a]

| | Serum deprivation[b] | | | Nocodazole[c] | | |
|---|---|---|---|---|---|---|
| | $G_1/G_0$ | S | $G_2/M$ | $G_1/G_0$ | S | $G_2/M$ |
| Untreated cells[d] | 50.7 ± 3.3 | 40.3 ± 2.3 | 9.1 ± 0.6 | 56.8 ± 2.0 | 37.5 ± 2.0 | 5.8 ± 1.1 |
| After synchronization[e] | 77.2 ± 3.6 | 8.1 ± 0.8 | 14.8 ± 2.9 | 13.0 ± 6.5 | 1.1 ± 1.5 | 86.0 ± 8.0 |
| 6 h after release[f] | 78.4 ± 2.6 | 9.8 ± 3.5 | 11.8 ± 1.2 | 56.2 ± 1.2 | 6.7 ± 3.7 | 37.1 ± 5.5 |
| 24 h after release | 68.4 ± 3.2 | 24.1 ± 3.4 | 7.5 ± 2.2 | 52.2 ± 3.0 | 19.5 ± 1.3 | 28.2 ± 4.3 |

[a]Cell cycle distribution was determined by flow cytometry after DAPI staining. The data show representative experiments done in duplicate. The whole experiment was repeated twice. Values are given as mean ± SD. [b]After plating, cells were cultivated for 24 h in the presence of 10% FCS. Thereafter, the cells were held for 48 h in serum-free medium. [c]After plating, cells were allowed to settle for 24 h in serum containing medium. Thereafter, the cells were treated for 16 h with nocodazole (0.5 µg $ml^{-1}$). [d]Cell cycle distribution 24 h after plating, immediately before synchronization. [e]After serum deprivation for 48 h or treatment for 16 h with nocodazole, respectively. [f]6 h after the end of the synchronization treatment by readdition of serum or removal of the nocodazole containing medium.

*© 2001 Cancer Research Campaign*

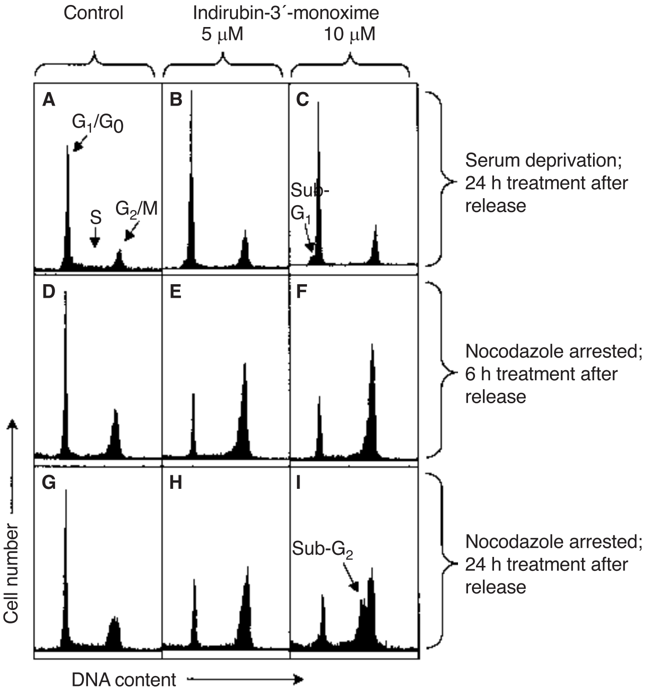

**Figure 4** Effect of indirubin-3′-monoxime on the cell cycle distribution of MCF-7 cells. (**A–C**) Cells were synchronized by serum deprivation (**A**) Control cells 24 h after readdition of serum. (**B**) 24 h incubation with 5 µM indirubin-3′-monoxime. (**C**) 24 h, 10 µM indirubin-3′-monoxime. (**D–I**) Cells were synchronized by nocodazole treatment. (**D**) Control cells 6 h after nocodazole treatment. (**E**) Incubation with 5 µM indirubin-3′-monoxime for 6 h. (**F**) 10 µM indirubin-3′-monoxime, 6 h. (**G**) Control cells 24 h after nocodazole treatment. (**H**) Incubation with 5 µM indirubin-3′-monoxime for 24 h. (**I**) 10 µM indirubin-3′-monoxime, 24 h. Cells were stained with DAPI and measured by flow cytometry. The charts show a representative experiment (*n* = 3)

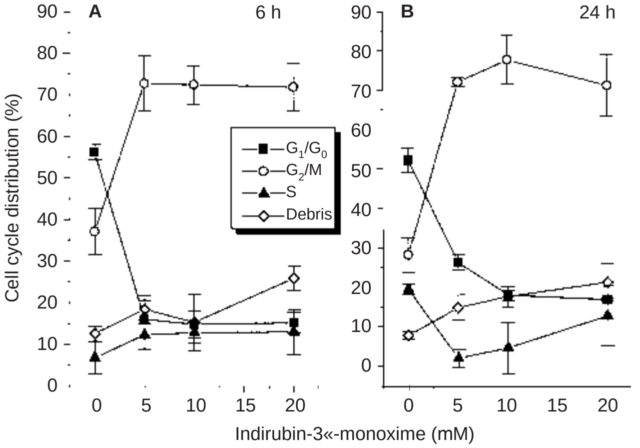

**Figure 5** Time-and concentration-dependent effect of indirubin-3'-monoxime on the cell cycle distribution of MCF-7 cells, synchronized by nocodazole treatment. Cells were treated for 6 h (**A**) and 24 h (**B**) with indirubin-3′-monoxime. After DAPI staining, cell cycle distribution was determined by flow cytometry. (■) $G_1/G_0$, (❍) $G_2/M$, (▲) S, (◊) debris. For each concentration, 2 Petri dishes were treated in parallel, the whole experiment was repeated twice. The values are given as mean ± SD

### Reduction of the active CDK1/cyclin B complex

Furthermore, we addressed the question whether treatment with indirubin-3′-monoxime influences the protein levels of cyclin B and CDK1 as well as the amount of cyclin B in complex with the kinase. MCF-7 cells were arrested by nocodazole treatment. After release, cells were incubated with 5 µM indirubin-3′-monoxime in serum-containing medium. For each time-point, a vehicle control was prepared in parallel. The total cellular content of cyclin B and CDK1 was determined by Western blot analysis using monoclonal antibodies. The chemoluminescent signal of the protein bands was quantified and calculated as percent of the respective vehicle treated control. After 1 h treatment, no significant changes in the total cyclin B and CDK1 protein levels were observed. The results after 3 h incubation indicate an increase of the protein levels of cyclin B and CDK1, but were not significant. By immunoprecipitation using a monoclonal anti-CDK1 antibody, followed by Western blotting with an antibody raised against cyclin B, we determined the amount of cyclin B in the active complex with CDK1. We observed a time-dependent change of cyclin B in complex with CDK1 induced by incubation with indirubin-3′-monoxime (Figure 7). Comparable to the results concerning the total amount of CDK1 and cyclin B, 1 h incubation resulted in a slight, not significant reduction of cyclin B. After 3 h of treatment, a significant increase of cyclin B in the active complex was detected

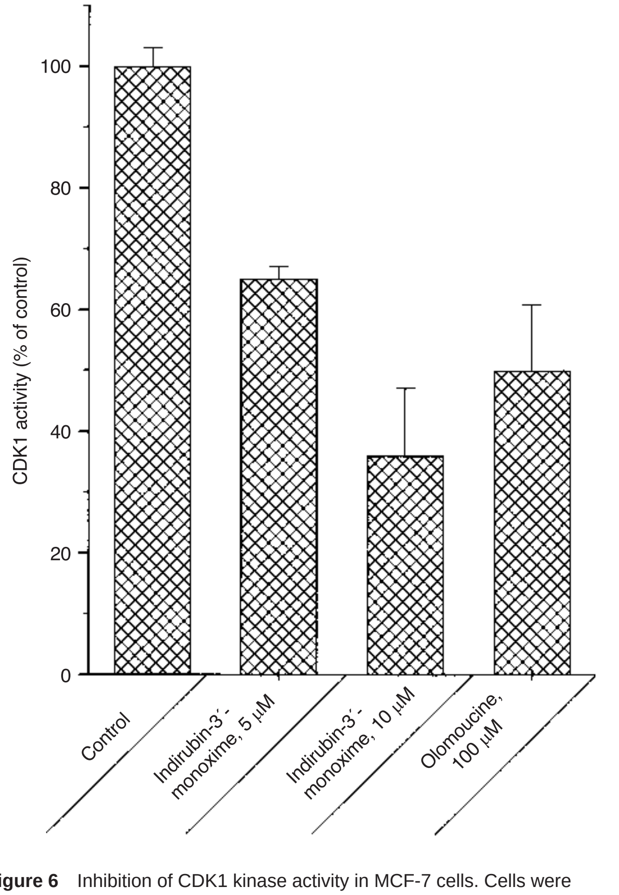

**Figure 6** Inhibition of CDK1 kinase activity in MCF-7 cells. Cells were synchronized by nocodazole treatment and subsequently incubated with the respective compound for 24 h. Thereafter, immunoprecipitation was performed using an antibody against human CDK1. Phosphorylation of histone H1 was determined using $^{32}$P-ATP. The CDK1 activity is expressed as % of the untreated control. The values are given a mean ± SD (*n* = 3)

© 2001 Cancer Research Campaign

($P < 0.05$). After 6 h incubation the total amount of cyclin B was significantly increased, whereas the amount of cyclin B in complex with CDK1 was significantly reduced ($P < 0.05$). A significant reduction of the total CDK1 protein was observed after 24 h treatment, paralleled by the reduction of cyclin B in complex with CDK1, whereas the total amount of cyclin B remained elevated.

### DISCUSSION

We have recently discovered, that indirubin-3′-monoxime potently inhibits cyclin-dependent kinases, such as CDK1/cyclin B, CDK2/cyclin A and CDK2/cyclin E (Hoessel et al, 1999). Since these observations were made using in-vitro kinase assays with isolated enzyme preparations, the question needed to be addressed whether CDK inhibition would also be seen in mammalian cells and whether this could be relevant for the growth inhibitory properties of indirubins. We here exemplify for CDK1 that indirubin-3′-monoxime inhibits CDK activity in MCF-7 cells at growth-inhibitory concentrations (Figure 6). Comparing the results of the cell cycle experiments (Figure 5) with the effects on CDK1 activity (Figure 6) demonstrates that despite the persisting $G_2$/M arrest of indirubin-3′-monoxime treated cells, expected to display high CDK1 activity in that cell cycle phase, in fact a dose-dependent inhibition of CDK1 activity is observed, compared to controls that have approached $G_1$. It appears unlikely therefore that the observed CDK1 activity inhibition is merely a reflection of changed cell cycle distribution.

Taking into account that the activity of isolated enzyme preparations of CDK1/cyclin B, CDK2/cyclin A, CDK2/cyclin E and CDK5/p35 are inhibited in the same concentration range (Hoessel et al, 1999), it can be expected, that in the cell in addition to CDK1 other CDKs are inhibited as well. This is supported by our findings that indirubin-3′-monoxime not only interferes with the release from $G_2$/M, as shown in cells arrested by nocodazole treatment, but also prevents $G_1$/S transition of cells synchronized in $G_1$/$G_0$ by serum depletion.

Concentrations of indirubin-3′-monoxime that induce CDK1 inhibition in cell culture are significantly higher, however, than those leading to the effective inhibition of isolated enzyme preparations. This reflects the potential influence of cellular uptake, subcellular distribution, elimination or metabolism. Indirubin and indirubin-3′-monoxime are taken up into cells to a similar extent. Despite significantly higher inhibitory potency of indirubin-3′-monoxime towards isolated CDK1/cyclin B kinase, indirubin-3′-monoxime exhibited only slightly enhanced growth inhibitory

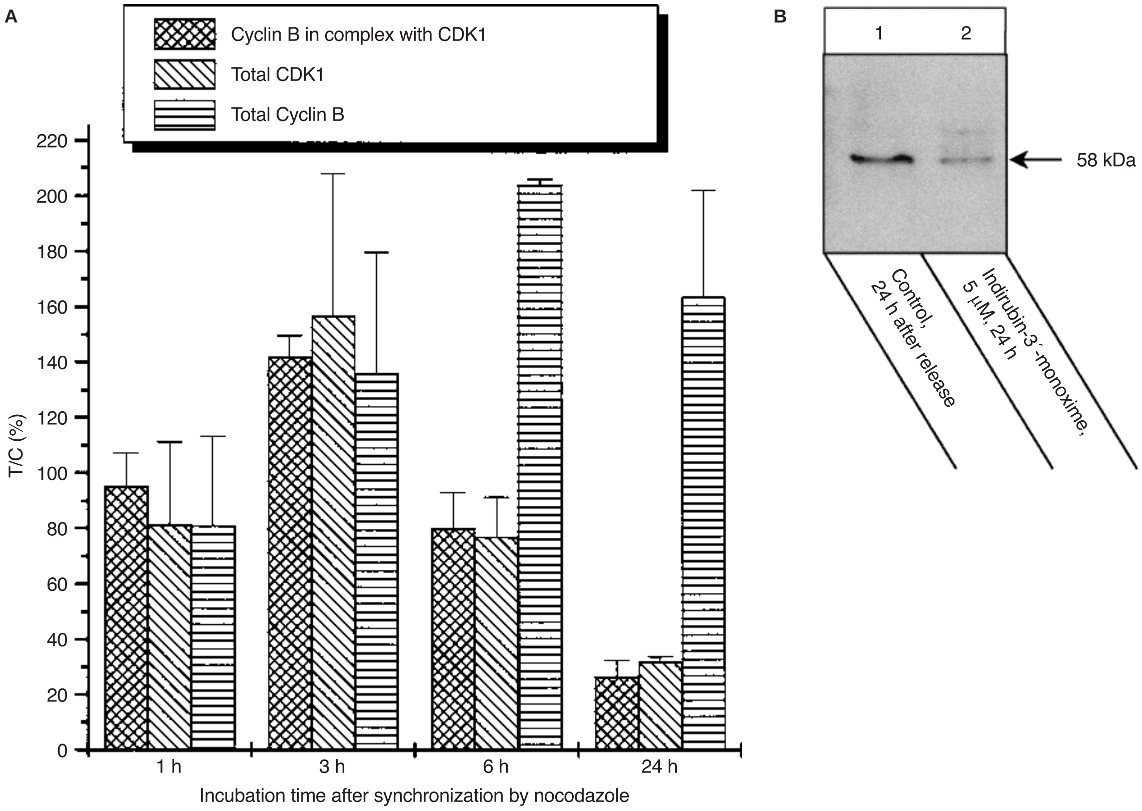

**Figure 7** (**A**) Total cellular content of cyclin B and CDK1 and of cyclin B in complex with CDK1 in MCF-7 cells, synchronized by treatment with nocodazole. The cells were incubated with 5 μM indirubin-3′-monoxime for 1 h, 3 h, 6 h and 24 h. Cell lysates were subjected to SDS PAGE (12% polyacrylamide) and transferred onto nitrocellulose. Western blotting was performed using monoclonal antibodies against human CDK1 or cyclin B, respectively. For detection of cyclin B in complex with CDK1, immunoprecipitation with a monoclonal antibody against CDK1 was followed by a Western blot using a monoclonal antibody against cyclin B. The protein levels are expressed as percent of the respective vehicle control at that time point. The values are the means ± SE of 3 independent experiments. (**B**) Immunoprecipitation (anti-CDK1), followed by Western blotting (anti-cyclin B) of MCF-7 cells 24 h after release from nocodazole treatment, incubated with 5 μM indirubin-3′-monoxime or vehicle control after synchronization. The figure shows a representative experiment, repeated twice

 © 2001 Cancer Research Campaign potency (Table 1). Apparently, differential subcellular distribution of the two compounds is of great additional influence. Whereas indirubin is predominantly localized in the particulate fraction, containing cellular membranes and nuclei, up to 44% of indirubin-3′-monoxime is detected in the cytosol. The crystal structure data of the indirubin-3′-monoxime complex with CDK2 show that the inhibitor binds to the ATP-binding site of the CDK subunit (Hoessel et al, 1999). The protein kinase CDK1/cyclin B plays a crucial role in the onset of mitosis in human cells. The CDK1/cyclin B1 complex accumulates in the cytoplasm during the $G_2$ phase, being translocated into the nucleus at the beginning of mitosis (Takizawa et al, 1999). Thus, for inhibitors targeting the CDK subunit, as it has been shown for indirubin-3′-monoxime (Hoessel et al, 1999), enhanced localization in the cytosol, might compromise the availability of the compound for inhibition in the relevant cellular compartment, the site of kinase activity: the nucleus.

The introduction of a sulfonate group into position 5 of indirubin potentiated the inhibition of isolated CDK1/cyclin B (Hoessel et al, 1999). However, the growth inhibitory properties are completely extinguished because this compound does not penetrate into the cell.

Furthermore, we investigated whether the inhibition of intracellular CDK1 activity is associated with changes in the expression of CDK1 or the regulatory subunit cyclin B. In MCF-7 cells arrested with nocodazole and treated with 5 μM indirubin-3′-monoxime after release, a significant increase of the cyclin B level was detected after 6 and 24 h. The enhanced cyclin B levels (compared to untreated control) appear to reflect the effective arrest of the indirubin-3′-monoxime treated cells in $G_2$/M (Figure 5), whereas control cells already move towards normal cell cycle distribution (Table 2). In contrast, after 24 h incubation, a significant decrease of the total CDK1 protein is observed, paralleled by the reduction of cyclin B in complex with CDK1 (Figure 7). At that time point, cells are still effectively arrested in $G_2$/M (Figure 5B), where high levels of the active CDK1/cyclin B complex are to be expected, compared to control cells already found predominantly in $G_1$ (Table 2). The cell cycle passage in mammalian cells is regulated by a complex system of activating and inhibiting factors. The different cyclin-dependent kinases act in distinct phases of the cell cycle. Thus, potent and selective inhibitors of CDKs might induce different effects on the cell cycle distribution, depending on the respective phase in which the cells are targeted. CDK1 and CDK2 play key roles at two different control points of the cell cycle. CDK2 is involved in the regulation of the $G_1$/S transition, whereas CDK1 is essential for the entry into mitosis. Our results suggest that indirubin-3′-monoxime interferes with cell cycle regulation at both restriction points. Olomoucine, an inhibitor of CDK1 and CDK2 kinases, has been reported to arrest cells both at the $G_1$/S and the $G_2$/M boundaries (Abraham et al, 1995).

We have previously shown, that treatment with low concentrations of indirubin-3′-monoxime arrests non-synchronized cells in the $G_1$ phase of the cell cycle and that at concentrations ≥5 μM an additional accumulation in $G_2$/M is observed (Hoessel et al, 1999). These data are consistent with our present results on the cell cycle effects in MCF-7 cells, synchronized by serum deprivation (Figure 3). In non-synchronized cells, the greatest proportion of the cells is in the $G_1$ phase. Synchronization by serum deprivation induces an arrest mainly in $G_1$, with some additional increase at $G_2$/M (Table 2). Thus, serum deprivation should pronounce effects of drugs acting at the $G_1$/S transition. In line with this argument, an increase of the cell population in $G_1$ at low substance concentration (2 μM) is observed after 24 h treatment with indirubin-3′-monoxime (Figure 3). Together with the data on non-synchronized cells (Hoessel et al, 1999), these results indicate that indirubin-3′-monoxime is active on CDK2 kinases interfering with the $G_1$/S transition in cell culture. However, at higher drug concentrations (≥5 μM) an additional accumulation in $G_2$/M has been observed (Hoessel et al, 1999). This is consistent with the present results showing indirubin-3′-monoxime induced inhibition of MCF-7 cells, arrested in $G_2$/M by nocodazole treatment, to further progress into $G_1$ (Figure 4 D–I, Figure 5). Moreover, after 24 h at 10 μM indirubin-3′-monoxime, a sub-$G_2$ peak became apparent, indicating the onset of apoptotic cell death (Figure 4 I).

Taken together, our results clearly show that indirubin-3′-monoxime exerts potent tumour cell growth inhibition and that this relates to inhibition of CDKs. As a result, progress through the cell cycle is blocked at $G_1$/S and $G_2$/M, events that might result in the induction of apoptosis.

### REFERENCES

Abraham RT, Acquarone M, Andersen A, Asensi A, Bellé R, Berger F, Bergounioux, C, Brunn G, Buquet-Fagot C, Fagot D, Glab N, Goudeau H, Goudeau M, Guerrier P, Houghton P, Hendriks H, Kloareg B, Lippai M, Marie D, Maro B, Meijer L, Mester J, Mulner-Lorillon O, Poulet SA, Schierenberg E, Schutte B, Vaulot D and Verlhac MH (1995) Cellular effects of olomoucine, an inhibitor of cyclin-dependent kinases. *Biol Cell* **83**: 105–120

Chinese Pharmacopoeia, Edition 1995, Vol. 1

Chen DH and Xie JX (1984) Chemical constituents of traditional Chinese medicine Qing Dai. *Chinese Trad Herbal Drugs* **15**: 6–8

Cooperative Group of Clinical Therapy of Indirubin (1980) Clinical studies of 314 cases of CML treated with indirubin. *Chinese J Intern Med* **1**: 132–135

Fiebig HH, Berger DP, Dengler WA, Wallbrecher E and Winterhalter BR (1992) Combined in vitro/in vivo test procedure with human tumor xenografts for new drug development. In: HH Fiebig and DP Berger (eds), Contrib. Oncol. Vol. 42: *Immunodeficient Mice in Oncology*, Basel: Karger, pp. 321–351

Hoessel R, Leclerc S, Endicott JA, Noble MEM, Lawrie A, Tunnah P, Leost M, Damiens E, Marie D, Marko D, Niederberger E, Tang W, Eisenbrand G and Meijer L (1999) Indirubin, the active constituent of a Chinese antileukemia medicine, inhibits cyclin-dependent kinases. *Nature Cell Biology* **1**: 60–67

Institute of Haematology, Chinese Academy of Medical Sciences (1979) Clinical studies of Dang Gui Lu Hui Wan in the treatment of CML. *Chinese Journal of Internal Medicine* **15**: 86–88

Marko D, Romanakis K, Zankl H, Fuerstenberger G, Steinbauer B and Eisenbrand G (1998) Induction of apoptosis by an inhibitor of cAMP-specific PDE in malignant murine carcinoma cells overexpressing PDE activity in comparison to their nonmalignant counterpart. *Cell Biochem Biophys* **28**: 75–101

Skehan P, Storeng R, Scudiero D, Monks A, McMahon J, Vistica D, Warren JT, Bokesch H, Kenney S and Boyd MR (1990) New colorimetric assay for anticancer-drug screening. *J Natl Cancer Inst* **82**: 1107–1112

Takizawa GG, Weis K and Morgan DO (1999) Ran-independent nuclear import of cyclin B1-cdc2 by importin β. *Proc Natl Acad Sci USA* **96**(14): 7938–7943

Wu LM, Yang YP and Zhu ZH (1979) Studies on the active principles of indigofera tinctoria in the treatment of CML. *Comm Chinese Herbal Med* **9**: 6–8

Wu GY, Fang FD, Liu JZ, Chang A and Ho YH (1980) Studies on the mechanism of action of indirubin in the treatment of chronic granulocytic leukemia. I. Effects on nucleic acid and protein synthesis in human leukemic cells. *Chinese Medical J* **60**: 451–454

Zheng QT, Lu DJ and Yang SL (1979) Pharmacological studies of indirubin. I. Antitumor effect. *Comm Chinese Herbal Med* **10**: 35–39

© 2001 Cancer Research Campaign
